# Health Effects of Grape Seed and Skin Extracts and Their Influence on Biochemical Markers

**DOI:** 10.3390/molecules25225311

**Published:** 2020-11-14

**Authors:** Lenka Sochorova, Bozena Prusova, Martina Cebova, Tunde Jurikova, Jiri Mlcek, Anna Adamkova, Sarka Nedomova, Mojmir Baron, Jiri Sochor

**Affiliations:** 1 Department of Viticulture and Enology, Faculty of Horticulture, Mendel University in Brno, Valtická 337, 691-44 Lednice, Czech Republic; tomaskova.l.9@gmail.com (L.S.); bozena.prusova@mendelu.cz (B.P.); MojmirBaron@seznam.cz (M.B.); 2 Centre of Experimental Medicine, Slovak Academy of Sciences, Institute of Normal and Pathological Physiology, 841-04 Bratislava, Slovakia; martina.cebova@savba.sk; 3 Faculty of Central European Studies, Institute for Teacher Training, Constantine the Philosopher University in Nitra, Drazovska 4, SK-949-74 Nitra, Slovakia; tjurikova@ukf.sk; 4 Department of Food Analysis and Chemistry, Faculty of Technology, Tomas Bata University in Zlin, Vavrečkova 275, 760-01 Zlin, Czech Republic; mlcek@utb.cz (J.M.); aadamkova@utb.cz (A.A.); 5 Department of Food Technology, Faculty of Agronomy, Mendel University in Brno, Zemedelska 1, 613-00 Brno, Czech Republic; sarka.nedomova@mendelu.cz

**Keywords:** antioxidants, cancer, cardiovascular disease, diabetes mellitus, gastrointestinal tract, grape seeds, grape skin, neuroprotective effect

## Abstract

This review is focused on the study of the effects of grape seed and skin extract (GSSE) on human health. GSSE contains high concentrations of important polyphenolic substances with high biological activity. This review is a summary of studies that investigate the effects of GSSE on diabetes mellitus, cardiovascular disease and cancer, its neuroprotective effect, and its effects on the gastrointestinal tract and other health complications related to these diseases. The results of the studies confirm that the anti-inflammatory, antiapoptotic, and pro-proliferative effects of “*Vitis vinifera* L.” seed extract reduce the level of oxidative stress and improve the overall lipid metabolism.

## 1. Introduction

The grapevine (*Vitis vinifera* L.) is an important cultivated plant that has long been studied due to its positive effects on consumer health. The seeds of this plant contain a wide range of biologically active components [1] that help to neutralize the adverse effects of free radicals. The high content of condensed flavan-3-ol or procyanidins, which are being investigated as one of the alternatives in the treatment of serious diseases, is particularly significant.

At the beginning of the 21st century, the health problems of developed countries rose to unbearable levels. The main causes of civilizational diseases include a lack of physical activity and poor lifestyle. With the growing awareness of diseases and the risks associated with them, more efforts are being made to prevent them. People are starting to take an interest in a healthy diet, and efforts toward maintaining mental and physical condition are improving [2]. New findings have highlighted insufficient intakes of substances that are contained mainly in plant components of food, which protect against the development of diseases other than cardiovascular disease and cancer [3]. These diseases are sometimes called “fruit and vegetable deficiency diseases.” [4]. According to the World Health Organization, these diseases are a major international problem and an important cause of cardiovascular disease and others. In recent years, there has been a growing interest in the study of biologically active components in fruits, vegetables, and other plant matrices.

Recently, the pharmaceutical and economic trends have focused on the search for new drugs in the field of secondary plant metabolites as substances for eliminating civilizational diseases. Thanks to the important biologically active components that the seeds of the grapevine (*Vitis Vinifera* L.) contain in their matrix, they can be used for medical purposes.

Grape seeds and skin are a significant source of polyphenolic substances (20–55%) [5]. Phenolic compounds contain a hydroxyl functional group (–OH) attached directly to a benzene ring (aromatic nucleus). Grape polyphenolics vary in chemical structure and activity and may be fundamentally categorized into two major classes: flavonoids and nonflavonoids. Flavonoids including anthocyanins, proanthocyanidins (procyanidins and prodelphinidins), and flavan-3-ols are distributed throughout the peel and seed [6]. In contrast, hydroxycinnamic acids, the most abundant non-flavonoids in wine, include caftaric acid and coutaric acid. The contents of individual polyphenolic substances depend on the grape variety [7].

A high content of proanthocyanidins in the seeds is rather important due to their positive effect on civilizational diseases [8,9,10,11]. Flavonoids contained in grapevine seeds show antibacterial and anxiolytic properties [12]. Nechita et al. [13] in their study on extracts originating from grapevine skins, seeds, grape pomace, and sediments mentioned their numerous pharmacological effects.

Grapevine seeds have high antioxidant potential; their beneficial effects include modulation of antioxidant enzyme expression, protection against oxidative damage in cells, anti-atherosclerotic and anti-inflammatory effects, and protection against certain types of cancer in both humans and animals. [14]

Almost 71.5% of the grapevine seed is dry matter. Water constitutes 28–44% of the total seed weight. However, grapevine seeds also contain a number of biologically active substances that, even in low concentrations, affect life processes, not only positively, but also negatively. These substances are isolated from natural sources, and they can differ significantly from each other in their structure. [15]

Numerous experiments have shown significant links between antioxidants, such as vitamins A and E and carotenoids (alpha-carotene, beta-carotene, xanthine, beta-cryptoxanthin, lycopene), and many acute diseases [16,17]. Grape seeds are a waste product in the wine sector, and the proper use of this waste product could lead to a new source of nutraceuticals. Vine seed oils are important common components of food, and growing evidence suggests that individual fatty acids from seeds may play different roles in human health, especially in the management of acute and chronic diseases [18,19]. Diets rich in specific fatty acids can potentially prevent many health problems or diseases. For example, omega-3 (n-3) unsaturated fatty acids help to prevent cancer, heart disease, hypertension, and autoimmune disorders. At present, consumers’ interest in improving their diet is increasing. These factors and others led to the development of the use of vegetable oils that have unique profiles of fatty acids and other useful ingredients, including phytosterols and natural antioxidants [20].

The novelty of this review is a comprehensive overview of the health effects of grape seed and skin extracts. Most civilizational diseases are caused by oxidative stress and inflammation, so the most important biomarkers, such as antioxidant activity, lipid peroxidation biomarkers, and anti-inflammatory biomarkers, have been studied.

The present study was based on a literature search in the databases Web of Science, PubMed, MEDLINE, Scopus, and Google Scholar. The findings of research studies chosen from more than 1000 viewed scientific publications were included/compared (about 85% of the articles were removed due to inconsistency regarding grape seed and skin extract (GSSE)). The search was based on keywords and phrases containing combinations of words such a biomarkers, bioactive compounds, phenolic composition of grapes, grape seeds, grape skin, and health effect. The present study primarily includes research findings from the years 2002–2020.

## 2. Studies Related to Diabetes Mellitus

Diabetes mellitus is a chronic disease that develops due to genetic predisposition and the presence of certain environmental factors. It is characterized by alterations in the metabolism of carbohydrates, fats, and proteins, which are caused by an absolute or relative lack of insulin secretion and varying degrees of insulin resistance. Excessive beta cell apoptosis is associated with the disease.

Grape seed extract (GSE) (*Vitis vinifera* L.) contains oligomeric proanthocyanidins (PACs), which show strong antioxidant activity. PACs have been able to control elevated levels of malondialdehyde (MDA) and carboxymethyllysine, reduce superoxide dismutase, and reduce glutathione (GSH) activity in mice with induced diabetes mellitus [8].

The results of an experiment conducted by Mansouri et al. [21] demonstrated that GSE can alleviate albuminuria and renal sclerosis in diabetic nephropathy. Feeding with GSE prevented the progression of diabetic nephropathy in rats due to its antioxidant properties [21]. The aim of a study conducted by scientists in 2011 [22] was to investigate the effect of GSE on oxidative stress in streptozotocin-induced diabetes in *Rattus norvegicus.* GSE was able to increase MDA levels and reduce plasma superoxide dismutase activity in rats with induced diabetes mellitus. It can be stated that GSE has the ability to alleviate oxidative stress by inhibiting lipid peroxidation and can restore endothelial function, thereby reducing the risk of vascular disease in patients with diabetes mellitus [23].

Grape seed oil was studied in the research of Professor Lai [24] from the University of China. The study was performed in vitro with *Rattus* pancreatic β-cells. and the oil proved to be promising due to its antidiabetic effects. The antiapoptotic activity of the oil was especially important. Cell apoptosis was detected by fluorescence-activated cell sorting, insulin secretion was detected by enzyme linked immunosorbent assay, and apoptosis genes were evaluated by quantitative reverse transcription of the polymerase chain reaction (qRT-PCR). The results of the experiment showed that grape seed oil, which contained 87% unsaturated fatty acids, was able to significantly reduce the apoptosis of pancreatic beta cells. Caspase-3, iNOS, ATF-3, JNK, p38, and Fas were “down-regulated,” while the anti-apoptotic genes Akt and Bcl-2/Bax were “up-regulated.” The results of the study showed that grape seed oil can protect pancreatic beta cells. Its protective activity may be associated with the mitochondrial pathways of the endoplasmic reticulum. Therefore, grape seed oil appears to be an effective dietary supplement or an alternative drug for patients with diabetes and leads to a reduction in apoptosis and dysfunction in beta cells [24].

An interesting study carried out by Professor Rashid’s team of researchers [25] showed that monomeric and oligomeric flavanols isolated from grape seeds were able to improve the condition of patients with microalbuminuria and had a positive effect on renal endothelial function in patients with diabetes mellitus.

A North Korean experiment [26] conducted in 2013 analyzed the neuroprotective effect of GSE in prediabetic mice. Mice were fed a high lipid diet. Immunohistochemical analyses were performed after 12 weeks. Intraepidermal innervation was quantified as the number of nerve fibers per unit length of the epidermis. This study provided scientific support for the therapeutic potential of the use of GSE in peripheral neuropathy. The results suggest that this extract could be used to treat diabetic peripheral neuropathy [26].

A histopathological study [27] conducted in 2018 investigated grape seed proanthocyanidin extract (GSPE). GSPE has a polyphenolic structure and has a wide range of biological activity. The aim of the study was to evaluate the effects of GSPE on alveolar bone loss and histopathological changes in 40 rats in which streptozotocin-induced diabetes mellitus and the ligation method induced periodontitis. Alveolar bone loss was measured morphometrically using a stereomicroscope. For histopathological analyses, alizarin red staining and matrix metalloproteinase, vascular endothelial growth factor immunohistochemistry, and inducible factor hypoxia were performed. Relative total inflammatory cells were also determined in tartrate acid-positive osteoclast cells. This animal study found that GSPE administration may reduce periodontal inflammation and alveolar bone loss by reducing alpha matrix metalloproteinase and hypoxia-inducible factor levels and increasing osteoblastic activity in diabetic rats with periodontitis [27].

Queslati et al. [28] study investigated the protective effect of grape seed and skin extract (GSSE) against diabetes-induced oxidative stress and renal dysfunction in rats. This study explored the effect of alloxan-induced diabetes on oxidative stress and renal dysfunction in virgin and pregnant rats, and the protective effect that a high dose of GSSE (4 g/kg) can provide. Diabetes adversely affected several parameters of renal function, such as creatinemia, uremia, uricemia, and proteinuria. Furthermore, it induced oxidative stress characterized by increased oxidation of lipids and proteins; decreased defense of antioxidant enzymes, such as catalase, superoxide dismutase, and glutathione peroxidase; altered transition metals, such as free iron, copper, selenium, and associated enzymes; and increased calpain and acetylcholinesterase. As a result, GSSE treatment had the ability to effectively protect against all the harmful effects of diabetes, such as renal dysfunction, in both virgin and pregnant animals. High-dosage GSSE is a safe and effective antioxidant that can be further tested in clinical trials for long-term maintenance of renal function, especially in multiple pregnancies [28].

In diabetes mellitus, serious secondary complications are nephropathy, neuropathy, and retinopathy. Patients with diabetes mellitus often develop nephropathy, a serious complication that has become a major cause of end-stage renal disease. An effective therapeutic strategy is being sought to prevent kidney damage in the terminal stage of the disease. In a study conducted by Zhang et al. [29] the protective effects of grape seeds, diabetic nephropathy, and complex quantitative proteomic analysis in mice with induced diabetes were investigated. The conclusion of the study was that oral administration of grape seeds significantly alleviated renal dysfunction and pathological changes in diabetic mice. The results of Western blot analysis confirmed that the milk fat of the EGF-8 globule was significantly regulated in the kidneys of mice with induced diabetes. This finding provided a new perspective on the pathogenesis of diabetic nephropathy.

A study [30] conducted in 2018 investigated the anti-inflammatory, antiapoptotic, and pro-proliferative effects of *Vitis vinifera L*. seed extract on the livers of rats with induced type 2 diabetes mellitus. Consumption of vine seeds has been found to improve the overall liver condition in rats with diabetes mellitus. However, the mechanisms on which its effects are based remain unknown. In this study, the anti-inflammatory, antiapoptotic, and pro-proliferative effects of ethanol extract from grape seeds on rat liver were found. Adult male rats with streptozotocin-nicotinamide-induced diabetes received 50, 100, or 200 mg/kg body weight of the extract orally for 28 days. At the end of treatment, body weight was determined and blood was collected for analysis of glucose, insulin, and liver enzyme levels. After sacrifice, the animals’ livers were removed and their weights and glycogen contents were measured. Histopathological changes in the liver were observed. The expression and distribution of inflammatory, apoptosis, and proliferative markers in the liver were identified using molecular biological techniques. Treatment of diabetic rats with ethanolic vine seed extract reduced body weight, liver weight, and liver glycogen content. In addition, fasting blood glucose and liver enzyme levels improved and serum insulin levels decreased. Moreover, minor histopathological changes were observed: reduction of inflammation and apoptosis, and reduction of inflammatory markers (TNF-alpha, nuclear factor-kappa (NF-k) beta, IKK-beta, interleukin-6 (IL-6), IL-1 beta) and markers of apoptosis (caspase-9 and Bax). Treatment with this ethanolic extract induces an increase in hepatocyte regeneration, indicating increased distribution of proliferating cell nuclear antigen and Ki-67 in the livers of diabetic rats. The conclusions highlight the anti-inflammatory, antiapoptotic, and pro-proliferative effects of the extract, which represent its hepatoprotective effects in patients with diabetes mellitus.

**Figure 1:**
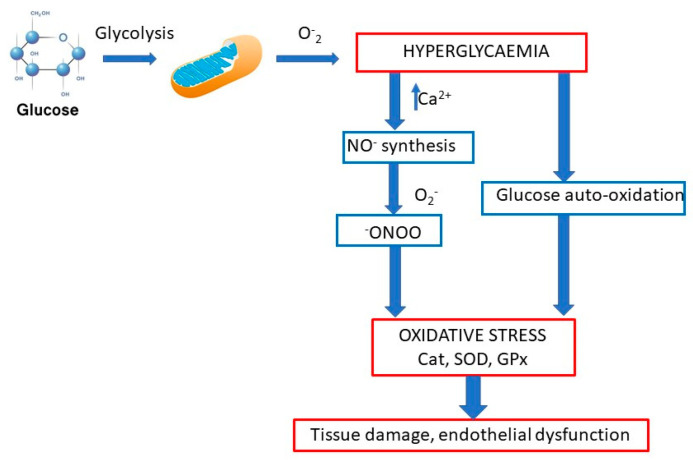
The primary causative factor of oxidative stress in diabetes mellitus is hyperglycemia. Higher glucose content leads to its oxidation, which causes tissue damage and endothelial dysfunction. Many studies have associated elevated serum calcium levels with markers of impaired glucose metabolism. In the presence of elevated calcium levels in endothelial cells, hyperglycemia stimulates the synthesis of NO^−^, which is in the presence of O^2•−^ converted to highly potent oxidant ^−^ONOO that promotes endothelial cell damage and endothelial dysfunction [31,32].

Oxidative stress and its associated hyperlipidemia and hyperglycemia play important roles in the development of diabetic nephropathy (Figure 1). Scientists Al-Malki et al. [8] studied the effects of PACs on the development of diabetic nephropathy in E-deficient mice. One group of mice was fed a “high-fat” diet (HFD) and the other HFD and PACs. High blood cholesterol levels were shown to be significantly reduced in the group of mice for which PACs were added to the diet. Furthermore, renal function was restored, and this led to a reduction in albuminuria compared with the untreated group. The administration of PACs increased levels of MDA and discarboxymethyllysine, reduced superoxide dismutase, and reduced GSH activity in diabetic mice [8].

Some polyphenols may potentiate the effects of insulin. Insulin transports glucose from the blood to skeletal muscle cells, the myocardium, and adipose tissue. The hormone causes exposure of the glucose transporters GLUT4 to the membrane. This happens in skeletal muscle cells, cardiomyocytes, and adipocytes. In this context, nerve centers important in the role of food intake; the roles of various neuropeptides; the neurohumoral effect on food intake; and the potential roles of polyphenols in influencing neuroregulatory factors, nerve signaling pathways, and/or peripheral feedback mechanisms that modulate food intake were studied [33].

Scientists [34] evaluated the inhibitory effects of plant extracts (including GSE) on alpha-amylase and alpha-glucosidase activity. These are important enzymes for starch digestion in humans. The results showed that GSE strongly inhibited both alpha-amylase and alpha-glucosidase activity. The experiment showed that plant extracts containing catechin 3-gallates, especially epigallocatechin gallate, are potent alpha-glucosidase inhibitors. Procyanidins in GSE strongly inhibit alpha-amylases.

Furthermore, natural polyphenols have been shown to alleviate dyslipidemia and ocular complications in rats with diabetes mellitus. In a study conducted by Baadescu et al. [35], polyphenols obtained from grape seeds were used and investigated in an experimental model (streptozotocin-induced diabetes mellitus) in *Wistar* rats. Streptozotocin was served in a single dose of 60 mg/kg of body weight. The extract was administered as an aqueous solution at a dose of 28 mg/kg body weight once every 2 days for 16 weeks. The obtained results indicate a significant improvement (*p* < 0.001) in the lipid profile and a reduced risk of atherogenicity. All experimental animals with induced diabetes had varying degrees of retinal detachment. Intake of polyphenolic extract in animals was able to alleviate retinal disease. Only accidental and uneven retinal detachments were observed [35].

The results of the study carried out by Iraq et al. [36] showed the effects of GSE on serum amylase levels and immunohistochemical changes in diabetic rats. The study examined serum amylase activity and structural changes in pancreatic tissue in rats. Amylase levels were found to be lowest in the diabetic group (794.00 ± 44.85 U/L, *p* < 0.001), while the GSE group had the highest value (1623.63 ± 80.04 U/L, *p* < 0.001). In the islets of Langerhans, an increased number of apoptotic cells was observed in the group of rats with diabetes. In the control and GSE groups, it was found that apoptotic cells were almost completely absent. In addition, atrophy in the islets of Langerhans, hyperemia in the capillary veins, and hydropic degeneration and necrosis of islet cells were found in the diabetic group. In the diabetes mellitus + GSE group, only mild hydropic degeneration was observed in the islets of Langerhans. In conclusion, it was found that GSE has a positive effect on the function and structure of the pancreas and improves the enzymatic activities and the structure of the islets of Langerhans.

The results of Mansouri et al.’s [21] experiment demonstrated alleviation of albuminuria and renal sclerosis in diabetic nephropathy with administration of GSE. Administration of the extract prevented the progression of diabetic nephropathy in rats due to its antioxidant properties [21].

Scientists Zhang et al. [37] investigated GSPE and its effects on alleviating urethral dysfunction in diabetic rats by modulating the nitric oxide-cyclic guanosine monophosphate (NO-cGMP) pathway. Oxidative stress is closely associated with the development of diabetes mellitus. Although diabetic urethropathy is one of the most common complications of diabetes mellitus, few studies have examined the role of oxidative stress in diabetic urethropathy. This study examined the role of oxidative stress and the protective effects of GSPE on urethral dysfunction using a streptozotocin-induced diabetes mellitus rat model. The findings of this study demonstrated that GSPE protects the urethral function in rats with diabetes mellitus by modulating the NO-cGMP signaling pathway. The protective roles of GSPE may be associated with the activation of the nuclear factor-erythroid 2-related factor 2 (Nrf2) defense pathway.

Intestinal alpha-glucosidases play a key role in carbohydrate digestion, and their inhibition provides a therapeutic option for suppressing postprandial blood glucose in diabetes [38]. An experiment showed that GSE significantly inhibited alpha-glucosidases. The effect of GSE was investigated as a potential antidiabetic agent. The extract was evaluated in an animal model of 6-week-old mice with streptozocin-induced diabetes mellitus. The results showed that the extract (400 mg/kg body weight) suppressed postprandial glycemia in diabetic mice. Oral administration of GSE reduced postprandial blood glucose levels in mice by 11.5% after 30 min and by 16.6% after 60 min. The results showed that the intake of GSE significantly reduced postprandial glycemia by 27.3% compared with the control, indicating that GSE could serve as an inhibitor of alpha-glucosidases in the future. The extract is therefore suitable for the prevention and treatment of diabetes [38].

Vascular complications in diabetes include endothelial dysfunction. The aim of the experiment conducted by Badavi et al. [39] was to determine whether the combination of the effects of GSE and exercise affected vascular endothelial function in *Rattus norvegicus* with induced diabetes. Forty-five male *Wistar* rats were divided into five groups. Responses to vasoactive substances (acetylcholine, phenylephrine, and sodium nitroprusside) were measured in experimental animals. The results showed weight loss in diabetic animals depending on the dose of vasoactive substance and exercise. Exercise in combination with intake of GSE increased plasma antioxidant capacity (AOC), lowered blood sugar levels, and restored vasodilatory responses to acetylcholine. Thus, the results showed the importance of taking GSE in combination with exercise rather than taking the extract alone. However, the vasoconstrictive response to phenylephrine and the vasodilatory response to nitroprusside were not significantly altered. Therefore, the combination of exercise and GSE had a significantly greater effect on endothelial dysfunction than exercise or GSE alone. This treatment could be an economically and ecologically acceptable therapy for vascular complications in people with diabetes [39].

Diabetes is associated with increased oxidative stress and impaired endothelium-dependent relaxation factor. This is the basis of many vascular complications. The aim of the experiment conducted by scientists from Turkey [22] was to monitor the effects of GSE on blood vessels and oxidative stress in streptozotocin-induced diabetes in rats. The rats were divided into three groups: a control group, a group of untreated diabetic rats, and a group of diabetic rats for which GSE was added at a dose of 100 mg/kg for 6 weeks. The results showed that GSE significantly increased MDA levels and reduced superoxide dismutase activity in plasma in rats. In conclusion, GSE can alleviate oxidative stress by inhibiting lipid peroxidation. In addition, it can restore endothelial function and reduce the risk of vascular disease in patients with diabetes [23]. An overview of diabetes studies is shown in Table 1.

## 3. Studies Related to Cardiovascular Disease

The goal of the team of Yamakoshi et al. [40] was to study a GSE rich in PAC, which reduced the development of aortic atherosclerosis in rabbits with high cholesterol. PAC-rich extracts (0.1% and 1% in the diet) did not significantly affect changes in the serum lipid profile of cholesterol-fed rabbits. The level of cholesteryl ester hydroperoxides induced by 2,2′-azobis (2-amidinopropane) dihydrochloride was lower in the plasma of rabbits fed the PAC-rich extract. There was a reduction in low density lipoprotein cholesterol and MDA when rabbits were fed with PAC-rich extract. Feeding PAC-rich extracts (0.1 and 1% in the diet) to rabbits significantly reduced severe atherosclerosis. Immunohistochemical analysis revealed a decrease in the number of oxidized LDL-positive, macrophage-derived foam cells in atherosclerotic lesions in the aorta of rabbits fed the extract. When the PAC-rich extract was administered orally, PAC was detected in plasma by the Porter method but not in lipoproteins (LDL plus VLDL). An extract rich in PAC was added in an in vitro experiment with human plasma. Plasma inhibited the oxidation of cholesteryl linoleate in LDL. These results suggest that PACs, the major polyphenols in red wine, may capture reactive oxygen species in plasma and interstitial fluid of the arterial wall, thereby inhibiting LDL oxidation and exhibiting anti-atherosclerotic activity [40].

Pinchuk and Lichtenberg [41] performed kinetic experiments to investigate the mechanism of action of antioxidants against lipoprotein peroxidation. Lipoprotein peroxidation in the blood is considered a key factor in the development of atherosclerosis. Therefore, attenuation of oxidative modification of lipoproteins by natural and synthetic antioxidants in vivo can be considered a possible way to prevent cardiovascular disease. Assessment of the susceptibility of lipoproteins to oxidation is commonly based on in vitro oxidation experiments. Oxidation monitoring provides a kinetic profile characteristic of a given lipoprotein preparation. The kinetic profile of peroxidation is characterized by three main parameters: the delay before rapid oxidation, the maximum oxidation rate (Vmax), and the maximum accumulation of oxidation products (ODmax). The addition of antioxidants changes this formula, which affects the kinetic parameters of oxidation. In particular, antioxidants may prolong the delay and/or decrease the Vmax and/or decrease the ODmax. Such a specific variation in a set of kinetic parameters may provide important information about the mechanism of the inhibitory effect of a given antioxidant (purification of free radicals, metal bonds, or other mechanisms). A number of natural and synthetic compounds inhibit the oxidation of lipoproteins. Based on the analysis of reported effects and theoretical considerations, a simple protocol was proposed that monitors the kinetic effects of a given antioxidant and its mechanism of action [41].

Etherton et al. [42] studied biologically active compounds in nutrition. They also focused on the methodology for determining biological functions and investigated the antioxidant and anti-inflammatory effects of flavonoids on atherosclerosis. Identification of bioactive substances and determination of their health effects are important areas of scientific research. There are promising prospects for selected bioactive compounds that reduce the risk of many diseases, including chronic diseases such as cardiovascular disease. Recent findings have shown that cardiovascular disease is inflammatory. Research has demonstrated that the adverse effects of oxidants on atherogenesis increase the possibility that antioxidants may have cardioprotective effects. Due to the number of bioactive compounds and the diversity of likely biological effects, numerous and diverse experimental approaches need to be adopted. Sophisticated experimental designs and analytical methods must be used to advance the field by identifying the complexity of their biology. The discovery of new effects of bioactive compounds on human health will provide a scientific basis for their future use as food ingredients, and in particular, as a means of improving public health [42].

**Figure 2:**
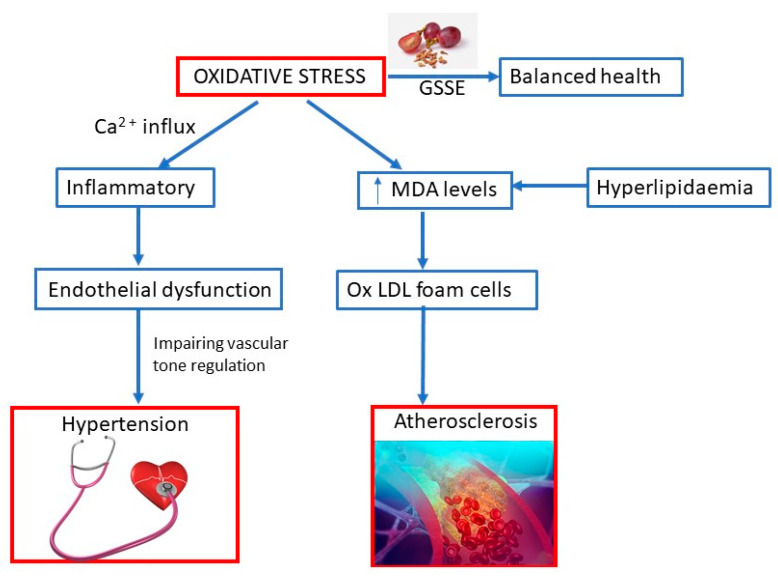
Oxidative stress causes inflammation, which leads to endothelial dysfunction, subsequently impairing vascular tone. Antioxidants in GSSE may act as scavengers for ROS, increase antioxidant enzymes, reduce oxidative and inflammatory process, and prevent endothelial dysfunction.

Oxidative stress may be induced by prooxidant stimulants, such as reactive oxygen species (ROS), AgII, and ET-1, and inflammatory cells that predominant over the antioxidant defense (Figure 2). Oxidative stress, inflammation, and endothelial dysfunction mutually amplify the damaging process. Oxidative stress causes inflammation, which leads to endothelial dysfunction, subsequently impairing vascular tone regulation and causing increased susceptibility to formation of foam cells and adverse vascular remodeling. Oxidative stress may cause Ca^2+^ influx in endothelial cells, which aggravates the inflammatory response, especially cell migration, and may cause vasodilation. Moreover, pro-oxidant and pro-inflammatory agent may mediate vascular smooth muscle cells’ Ca^2+^ influx, later causing vasoconstriction. Antioxidants may act as scavengers for ROS, increase antioxidant enzymes, reduce oxidative and inflammatory processes, improve endothelium-derived hyperpolarizing factor and baroreflex sensitivity, and prevent endothelial dysfunction [43].

An experiment performed by Aldini et al. [44] examined procyanidins from grape seeds, which were found to protect endothelial cells from peroxynitrite damage and improve endothelial relaxation in the human artery. This provided new evidence for cardioprotection. Peroxynitrite’s ability to capture procyanidins from *Vitis vinifera L*. seeds was studied in a homogeneous solution and in ocular endothelial cells (EA hybrid cell line) using 3-morpholinosydnonimine as a peroxynitrite generator. When endothelial cells were exposed to 5 mM 3-morpholinosydnonimine, significant morphological changes indicating necrotic cell death were observed (cell viability reduced to 16 ± 2.5%). Cell damage was suppressed with procyanidin with a minimum effective concentration of 1 µM (overall cell morphology and integrity were restored at 20 µM). The cellular localization of procyanidins in endothelial cells was confirmed using a new staining procedure and site-specific peroxyl radical inducers. Endothelial cells preincubated with procyanidins (20 μM) and exposed to FeCl_3_/K_3_Fe(CN)_6_ showed a characteristic blue staining index of site-specific binding of procyanidins to endothelial cells. Procyanidins dose-dependently inhibited lipid oxidation and exacerbated the consequent loss of cell viability, but were ineffective when oxidation was conducted at the intracellular level. This demonstrates that the protective effect is due to their specific binding to the outer surfaces of endothelial cells, thereby quenching exogenous harmful radicals [44]. An overview of cardiovascular studies is shown in Table 2.

## 4. Studies Related to Oncological Diseases

**Figure 3:**
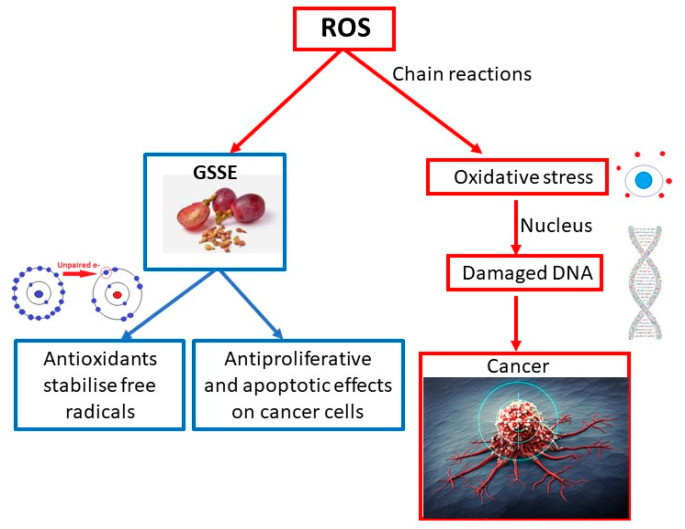
The presence of free radicals leads to uncontrollable chain reactions, causing oxidative stress. Free radical attacks on nuclei lead to mutations or DNA damage, which in turn, lead to cancer. Many antioxidants obtained from grapevine seeds help to scavenge free radicals and convert them to a stable, non-reactive form.

In a study led by Professor Nirmala [45], cytotoxicity and apoptotic cell death induced by extracts from the shells and seeds of *Vitis vinifera L*. on A431 skin cancer cells were investigated. The grape is one of the most widespread fruits in the world and is rich in antioxidant polyphenols. This study was conducted to assess the antiproliferative and apoptotic effects of GSSEs in an in vitro model using A431 human epidermoid carcinoma cell lines. Extracts from the seeds and husks of *Vitis vinifera L*. were incubated with A431 cells to evaluate antiproliferative and apoptotic effects and morphological apoptotic changes. Mitochondrial membrane potential was also measured after incubating the cells with the extracts. At the inhibitory concentration (IC50), the grape extract (111.11 µg/mL) was incubated with A431 cells for 24 h. GSSEs significantly affected cytotoxic effects and induced apoptosis and apoptotic morphological changes in A431 cells (*p* < 0.01); this effect is associated with interference with mitochondrial membrane potential. This decrease in mitochondrial membrane potential probably initiated an apoptotic cascade in treated cells. Phytochemicals from *Vitis vinifera L*. can selectively target cancer cells and serve as potential antitumor agents, providing better efficacy in killing cancer cells [45]. The mechanism of cancer development and the effect of GSSE on the prevention and treatment of this disease is shown in Figure 3.

Drisko et al. [46] studied antioxidants that reduce the negative impact of chemotherapy. They aimed to determine whether they also reduce its effectiveness. Antioxidants have a cytotoxic effect and can induce apoptosis in cancer cells. Increased production of free radicals occurs during chemotherapy and radiation therapy. Most cancer cells had elevated catalase during antioxidant treatment. It decomposes hydrogen peroxide into oxygen and water [46].

Adriana et al. [47] studied the effects of grape extract on breast cancer. Mare de Recas GSE was evaluated for its antitumor activity against Ehrlich ascites carcinoma in mice. The amount of GSE polyphenols, expressed as gallic acid, was 11.477 mg/L. The antitumor activity of GSE was determined by measuring tumor volume and counting tumor cells. Total thiobarbituric reactive substances (TBARS) in plasma were measured as parameters of oxidative stress. The results show that GSE is not effective against cancer and does not interfere with the antitumor effects of doxorubicin in mice but reduces lipid peroxidation induced by doxorubicin treatment.

In an experiment conducted in 2017 [48], the chemopreventive potential of blue grape marc powder against colorectal cancer in HT-29 cells was tested. This study evaluated the antiproliferative and antigenotoxic effects of grape marc powder (seedless variant, seeds alone). In vitro gastrointestinally digested and fermented fractions were used in cell treatment. Phenolic acids from seedless compacts showed the highest bioavailability in the small intestine, while the polyphenols contained in grape seeds may be most fermentable in the large intestine. Dietary fiber from seedless compacts was the best substrate for the production of short-chain fatty acids in the intestinal microflora. The viability of colon cancer cells (HT-29) was inhibited by 50% (IC50 values) at treatment concentrations ranging from 845 g/mL (seedless compacts) to 1085 g/mL (seeds) before digestion, and all digested fractions showed similar antiproliferative effects (mean IC50 = 814 g/mL). Oxidative DNA damage in cells was also attenuated by treatment (200 μg/mL, 24 h before incubation), with all fractions showing similar genoprotective effects. These results demonstrate the chemopreventive potential of grape marc powder in colorectal cancer [48].

A study carried out in 2018 [49] focused on the biochemical evaluation of selected grape varieties and grape extracts (*Vitis vinifera* L.) grown in Jordan, used against human prostate cancer cells. Sixteen different varieties collected in Jordan were evaluated. The validated liquid chromatography electrospray ionization tandem mass spectrometric method was used for the quantitative analysis of resveratrol in grape extracts and in the grapes themselves. GSEs showed higher contents of resveratrol than seeds. Antioxidant activity and total phenol content in extracts from seeds, husks, and whole berries of varieties were evaluated using the DPPH method and the Folin–Ciocalteu colorimetric method. Seed extracts showed the highest total phenol content and antioxidant activity. The in vitro effects of Golden Scatt GSE on prostate cancer cell migration were tested. Golden Scatt GSE inhibited the migration potential of prostate cancer cells in a dose-dependent manner.

Grapes contain high concentrations of PACs, which are important in the prevention of many types of cancer. The aim of an experiment [50] was to analyze extracts from grape seeds and compare their antiproliferative effects on the most common type of oral cancer. Tests were performed using two squamous cell lines (CAL27 and SCC25) to evaluate the effect of GSE. GSE exerted different effects on cell adhesion, cell morphology, and cell cycle regulatory pathways. This study confirms that GSEs inhibit the spread of oral cancer and that the mechanism of this inhibition may work on the principle of activating key apoptotic regulatory mechanisms in these cell lines.

Kaur et al. [51] investigated GSE and its ability to induce apoptosis in colorectal cancer (CRC) cells. One of the preventive measures of CRC is the intake of dietary substances or food supplements. As most of these products are often perceived by patients as complementary or alternative medicine, their effectiveness needs to be confirmed, the mechanism of action clarified, and the preparation standardized. GSE is one such supplement that people often consume due to its many health benefits. It has recently been confirmed that GSE inhibits the growth of HT29 cells in CRC cells in mice. Since GSE is commercially available through various suppliers, an experiment was conducted to evaluate whether GSE from two different manufacturers produced comparable biological effects in human CRC cell lines. The results show that regardless of the source, GSE strongly inhibits the growth of LoVo, HT29, and SW480 cells with G1 arrest in LoVo and HT29 cells. GSE also induced Cip/p21 levels in all three cell lines. In addition, induction of apoptosis by GSE was observed in all three cell lines. The findings suggest that GSE may be an effective anti-CRC agent due to its strong inhibitory effects [51].

A study conducted by Xue et al. [52] tested the effect of grape procyanidin extract against lung cancer, and its bioavailability and bioactivity. MiR-106b is a potential target for antitumor therapy. It was believed that GSPE has antineoplastic effects on lung cancer through modulation of miR-106b and its subsequent target. It was found that GSE significantly reduced MiR-106b levels in various lung neoplastic cells and increased levels of mRNA and protein (p21) of the 1A inhibitor kinase inhibitor (CDKN1A). MiR-106b transfection mimics the rearrangements of CDKN1A mRNA and p21, abolishing GSE-induced antiproliferative and anti-invasive properties in lung cancer cells. Oral gastric nutrition of leucoselect phytosome (LP), standardized expression of MiR106B mRNA and MiR-106b in GSE expression of athymic mice, and increased expression of CDKN1A mRNA in tumor xenografts correlated with a significant reduction in tumor growth. To assess bioavailability, GSE and metabolites in plasma levels were measured at weeks 4 and 8 of treatment 60–90 min after the LP probe. The findings show new antineoplastic mechanisms of GSE, further define the pharmacokinetics and pharmacodynamics of LP, and support further research on the use of LP against lung cancer [52].

A study found that PACs from grape seeds inactivated the PI3-kinase/protein kinase B pathway and induced apoptosis in a colon cancer cell line. The aims of this study [53] were to evaluate the chemopreventive/antiproliferative potential of GSPE against colon cancer cells (CaCo2 cells) and to investigate the mechanism of its action. GSPE (10–100 g/mL) significantly inhibited cell viability and increased CaCo2 cell apoptosis but did not alter viability in the normal cell line (NCM460). The increased apoptosis observed in GSPE-treated CaCo2 cells correlated with PI3-kinase attenuation (p110 and p85 subunits) and decreased PKB Ser473 phosphorylation. GSPE apparently has positive effects, increases apoptosis, and suppresses important pathways associated with PI3-kinase survival [53].

In 2018, Hamza et al. [54] performed a molecular characterization of the effect of GSE against chemically induced liver cancer. The purpose of this study was to investigate the anticarcinogenic properties of GSE in the early stages of liver cancer using two-stage treatment. GSE was administered at doses of 25, 50, and 100 mg/kg daily for 14 weeks. GSE dramatically inhibited the formation of pre-neoplastic foci, and significantly reduced the amount of placental glutathione-S-transferase in the livers of DEN-2AAF-treated rats by between four and ten times. The effects of GSE have been associated with induced apoptosis; decreased cell proliferation; decreased oxidative stress; and decreased regulation of histone deacetylase activity and inflammation—cyclooxygenase 2, inducible nitric oxide synthase, nuclear factor-kappa B-p65, and β-phosphorylated tumor necrosis factor (TNF) receptors in the liver. GSE treatment also reduced the viability of HepG2 cells and induced early and late apoptosis by activating caspase-3 and Bax. In addition, GSE induced G2/M and G1/S cell cycle arrest. This study provides evidence that the antitumor effect of GSE is mediated through inhibition of cell proliferation, induction of apoptosis, modulation of oxidative damage, and suppression of the inflammatory response [54].

A study conducted in 2018 [55] found that Autumn Royal and Ribier grape juice extracts (GJEs) reduced the viability of colon cancer cells and their potential to metastasixe. This study evaluated the chemopreventive and antitumor properties of phenolic compounds present in Autumn Royal and Ribier GJEs. The effects of these GJEs on the viability (daily sulphorodamine analysis) and metastatic potential (migration and invasion parameters) of HT-29 and SW-480 cell lines were evaluated. The effects of GJE on the two matrix metalloproteinase gene expressions (MMP2 and MMP9) were evaluated by qRT-PCR. In the first case, GJE reduced cell viability in both cell lines in a dose-dependent manner. GJE treatment also reduced cell migration and invasion. The results provide new information on the antitumor properties of selected GJEs in colon cancer cells [55].

Grape seed proanthocyanidins (GSPs) inhibit the proliferation, migration, and invasion of lung cancer cells by suppressing the protein kinase B/nuclear factor-kappa B signaling pathway. Tissue cell carcinoma (TSCC) is the most common oral cancer. Despite significant therapeutic progress, the five-year survival rate of patients with TSCC has not improved; this is caused by regional recurrences and metastases to the lymph nodes. GSPs are consumed worldwide as dietary supplements that show antitumor activity against several different types of cancer. However, their impact on TSCC and the basic mechanism of their functioning remains unclear. In this study, GSPs were found to significantly inhibit viability and induce dose-dependent apoptosis of Tca8113 cells. This was associated with significantly increased BAX pro-apoptosis regulator protein expression and significantly decreased Bcl-2 anti-apoptosis regulator protein expression at a dose of 100 μg/mL GSP. In addition, at non-toxic concentrations, GSPs significantly inhibited the secretion of matrix metalloproteinase-2 (MMP-2) and MMP-9 from Tca8113 cells, and their migration and invasion. Furthermore, GSPs were shown to significantly inhibit the phosphorylation of protein B kinase B (Akt) and kappa B I, and the translocation of nuclear factor-kappa B (NF-kappa B) into the nucleus of Tca8113 cells. Taken together, these results suggest that GSPs inhibit the proliferation, migration, and invasion of Tca8113 cells by suppressing the Akt/NF-kappa B signaling pathway. This suggests that GSPs may be developed as new potential chemopreventive agents against TSCC [56].

Studies have shown that a diet rich in fruits and vegetables can reduce the incidence of tumors. In particular, GSE has been shown to have chemopreventive and antitumor activities due to the many useful substances it contains. A study carried out by Iannoe et al. [57] characterized the anticancer properties of GSEs rich in chitosan-microencapsulated extracts in vitro. The purpose of this study was to create a biocompatible matrix containing GSE to obtain microparticles capable of modulating its biopharmaceutical parameters. The spray drying technique was chosen to realize chitosan microparticles characterized by a mean diameter of 4–10 μm and a positive surface charge. Evaluation of the pharmacological activities of GSE and chitosan-containing microparticles on various cancer cells showed an increase in antitumor effect due to increased cell interaction. Therefore, chitosan microparticles containing GSE may be an innovative system that is useful for the treatment of some cancers. [57] An overview of oncological studies is shown in Table 3.

## 5. Studies Related to the Neuroprotective Effect

**Figure 4:**
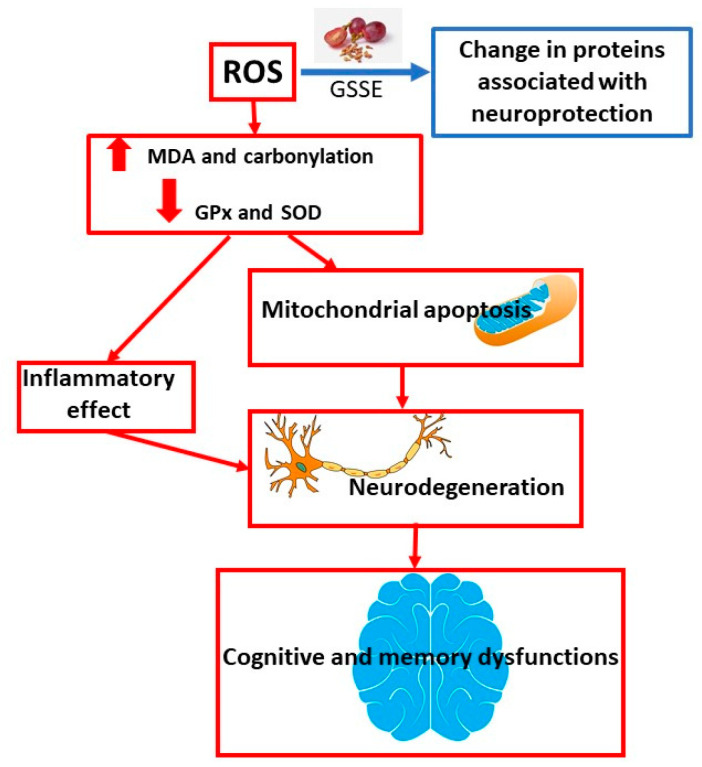
ROS has a negative effect on the life of brain cells, which can lead to neurodegenerative disorders and reductions in cognitive and memory functions. The antioxidants contained in GSSE can positively affect the amount of ROS and convert them to less reactive forms.

An experiment conducted by Singh et al. [58] investigated the neuroprotective effects of resveratrol. Resveratrol, a natural stilbene present in relatively high concentrations in seeds, husks, and red wine, is known for its antioxidant activity in the vascular and nervous systems. In contrast to its direct antioxidant role in the central nervous system, recent research identified a protective mechanism through increased endogenous cellular antioxidant defense that triggers a cascade of parallel neuroprotective pathways. A growing body of in vitro and in vivo evidence suggests that resveratrol acts in multiple ways and reduces ischemic damage to vital organs such as the heart and brain. Most of the protective biological effects of resveratrol are associated with its antioxidant, anti-inflammatory, and antiapoptotic properties and other indirect pathways. The continuing public interest and the growing availability of resveratrol on the market require an overview of available in vitro and in vivo experiments. There is a lack of rigorous clinical trials evaluating the effects of resveratrol in stroke. Resveratrol has shown potential for the treatment of stroke in laboratory animals and in in vitro studies in human cells; however, there is still a need for research in preclinical conditions. This review summarizes much of the knowledge about the neuroprotective potential of resveratrol in stroke with a focus on experimental in vitro and in vivo models and on some proposed mechanisms of action [58]. The mechanism of neurodegeneration development and the overall effect of GSSE on the prevention and treatment of this disease is shown in Figure 4.

Kim et al. [59] analyzed the effects of GSE on the brain of rats and focused on the technological and biological consequences of psychoactive substances. It was hypothesized that the use of polyphenol-rich GSE would result in a change in proteins associated with neuroprotection. Proteomics technologies, namely, 2D gel electrophoresis and mass spectrometry, have identified quantitative changes in specific proteins induced in the rat brain after ingestion of GSE. In conclusion, GSE has a neuroprotective effect by affecting specific proteins. This study discusses elements of proteomic analysis that demonstrate the power of technology to detect proteins that are involved in the brain’s response to a stimulus, whether a nutritional supplement or a psychoactive drug. In addition, the fact that GSE affects proteins implicated in cognitive disorders suggests that GSE may have impacts on the effects of psychoactive drugs by maintaining the overall viability of the nervous system [59].

In a study conducted in 2018, Yousef et al. [60] explored the neuroprotective and nephroprotective effects of GSPE on the modulation of inflammation, the tumor suppressor protein p53, neurotransmitters, oxidative stress, and histology. The combination of thalidomide and carboplatin is one of the most effective chemotherapeutic strategies for the treatment of cancer. However, few neurotoxicity and nephrotoxicity studies of both chemotherapeutic agents have been conducted. The aim of the study was to evaluate the toxicity of the combination of thalidomide and carboplatin on the brain and kidneys and to examine the protective effect of GSPE. Thalidomide and carboplatin induced increased regulation of the expressions of p53, TNF-alpha, and IL-6 in the brain and kidney. Acetylcholinesterase, dopamine, and serotonin decreased, and norepinephrine increased. Reagents with thiobarbituric acid, nitric oxide, lipid profile, bilirubin, and creatinine were increased, while antioxidant enzymes (GST, glutathione peroxidase (GPx), catalase (CAT), and superoxide dismutase (SOD)), total AOC levels, and GSH levels were reduced. Microscopic examination showed capillary shrinkage, degeneration with pyknotic nuclei, loss of normal structure, and neuronal degeneration. Concomitant treatment of GSPE with thalidomide and carboplatin reduced brain and kidney damage; reduced oxidative stress; reduced cytokines, p53, neurotransmitters, and biochemical parameters; and inhibited brain and renal cell apoptosis. It can be stated that the protective effects of GSPE against thalidomide and carboplatin-induced brain and kidney damage have been associated with the minimization of oxidative stress [60].

Dementia is a risk factor accompanying obesity. Fat can adversely affect the brain. GSSE can provide protection against high lipid dietary steatosis and increased oxidative stress. A diet high in lipids is associated not only with brain lipotoxicity, but also with increased oxidative stress, which is characterized by increased lipoperoxidation and carbonylation and inhibition of glutathione peroxidase and superoxide dismutase. The results showed that the extract mitigated all the harmful effects of HFD. Grape seed and husk extract could be used as a safe preparation against lipotoxic agents and as a prevention against brain damage caused by higher doses of lipids [11]. An overview of neuroprotective studies is shown in Table 4.

## 6. Studies Related to the Gastrointestinal Tract

In a study carried out by Oteiza et al. [61], flavonoids and their local and systemic effects on the gastrointestinal tract were investigated. The gastrointestinal tract plays a central role in the absorption, distribution, metabolism, and excretion of flavonoids, which ultimately define the health effects of these bioactive substances. These aspects are modulated by the interaction of flavonoids with other components of the diet, environmental factors, and hosts and microorganisms of the gastrointestinal tract. Flavonoids can target molecules in the luminal contents of various cell types of the gastrointestinal tract. Importantly, flavonoid effects on the gastrointestinal tract can have systemic effects on such thing as glucose homeostasis, lipid and energy metabolism, and cardiovascular factors. The beneficial effects of flavonoids on the gastrointestinal tract include their ability to protect the intestinal epithelium against pharmacological and food toxins; modulate the activity of enzymes involved in the absorption of lipids and carbohydrates; maintain the integrity of the intestinal barrier; modulate intestinal hormone secretion; modulate the immune system of the gastrointestinal tract; develop potential colorectal anticarcinogenic activity; and shape the composition and function of microorganisms in the intestine. It is crucial to gain an understanding of the mechanisms of mediating the effects of flavonoids on the intestine (and its microflora) given the importance of the gastrointestinal tract for maintaining overall health [61].

A study conducted by Pinentem [62] investigated the antioxidant effects of natural extracts rich in PAC from grape seeds and grapefruit on the gastrointestinal mucosa. The gastrointestinal tract is constantly exposed to free radicals released by the gastrointestinal tract itself and to free radicals present in food and beverages. Phenolic compounds can help to protect the gastrointestinal tract from damage caused by free radicals. The effects of GSPE on oxygen free radical production (ROS) in two different intestinal cell types, the CaCo-2 absorption cell line and the STC-1 enteroendocrine cell line, were analyzed. The results show that GSPE prevents the oxidative stress induced by tert-butyl hydroperoxide in both cell lines and that the effects are dose- and time-dependent. Analysis of whether GSPE had an in vivo effect found that 25 mg/kg body weight could not rule out an increase in intestinal ROS induced by poor lifestyle. However, an acute (1 h) dose of 1 g GSPE/kg body weight reduced ROS in fasted animals and reduced the induction of ROS by food. These effects have been observed with short-term treatment. Furthermore, the in vitro effects of GSPE were compared with the effects of other PAC-rich extracts, such as cupuassu seeds. Cupuassu extract has antioxidant effects in both cell types, suggesting different mechanisms from GSPE. In conclusion, natural extracts rich in PAC have an antioxidant effect in the gastrointestinal tract by acting on absorption cells and enterohormone-secreting cells, although the effects depend on the dose and duration of treatment [62].

Sanchez-Patan’s study [63] compared in vitro microbial fermentation in cranberry and grape extracts. The extracts were used directly without prior intestinal digestion. Of the 60 target phenolic compounds, our results confirm the formation of phenylacetic, phenylpropionic, and benzoic acids; and phenols, such as catechol and its derivatives, via the action of microbial tissue on polyphenols. Benzoic acid (38.4 g/mL), 4-hydroxy-5-(3′-hydroxyphenyl) valeric acid (26.2 g/mL), and phenylacetic acid (19.5 µg/mL) reached the highest concentrations. Under the same conditions, there was less microbial degradation of grape polyphenols compared with cranberry polyphenols, which was consistent with the more pronounced antimicrobial effect observed with grape polyphenols, especially against *Bacteroides*, *Prevotella*, and *Blautia coccoides-Eubacterium rectale* [63].

An experiment on GSE, which reduces oxidative stress and fibrosis during experimental biliary obstruction, was conducted by Dulundu et al. [64]. The aim of the study was to assess the protective effect of GSE against oxidative liver damage and fibrosis caused by biliary obstruction in rats. GSE was administered orally at a dose of 50 mg/kg daily for 28 days. Aspartate aminotransferase (AST), alanine aminotransferase (ALT), and lactate dehydrogenase (LDH) levels were determined to assess liver and tissue damage. TNF-alpha and AOC were tested in plasma samples. Liver tissues were collected to assess liver MDA and GSH levels, myeloperoxidase (MPO) activity, and collagen content. The production of reactive oxidants was monitored by a chemiluminescence assay. The results showed that serum AST, ALT, LDH, and plasma TNF-alpha were increased in the biliary obstruction group compared with the control group and were significantly reduced with GSE treatment. The increases in tissue MDA levels, MPO activity, and collagen content due to biliary obstruction were attenuated by GSE. In addition, luminol and lucigenin levels in the biliary obstruction group increased dramatically compared with the control group and were reduced by GSE treatment. These results suggest that GSE protects the liver from oxidative damage after bile duct ligation in rats. This effect is likely to include inhibition of neutrophil infiltration and lipid peroxidation, thereby restoring the oxidative and antioxidant state in the tissue [64].

In a study conducted by Cires et al. [65] PACs in the gastrointestinal tract were tested as the main target organ for promoting health effects. PACs are polymers of flavan-3-ol that are consumed in large amounts in the human diet. There is growing evidence to support the beneficial effects of PACs on the prevention of chronic diseases. It is believed that PACs with a degree of polymerization greater than three remain unabsorbed in the gastrointestinal tract and accumulate in the intestinal lumen. Accordingly, the gastrointestinal tract can be considered a key organ for the health benefits of PACs. PACs form non-specific complexes with salivary proteins in the mouth, which evoke a feeling of astringence. They also have antimicrobial effects that disrupt cariogenic or ulcerogenic pathogens in the mouth (*Streptococcus mutans*) and stomach (*Helicobacter pylori*). Through the antioxidant and anti-inflammatory properties of PACs, they reduce the inflammatory processes of gastrointestinal inflammation. Interestingly, they exhibit prebiotic activity and thus stimulate the growth of *Lactobacillus spp*. and *Bifidobacterium spp*. and some butyrate-producing bacteria in the large intestine. PACs are also metabolized by the intestinal microflora, which produce metabolites, especially aromatic acids and valerolactones, which accumulate in the large intestine and/or are absorbed into the bloodstream. Accordingly, these compounds could exhibit biological activities on the colon epithelium or in extra-intestinal tissues [65].

In an experiment conducted in 2018, Chede et al. [66] studied the intestinal absorption and antioxidant activity of polyphenols from grape marc. The absorption and antioxidant activity of polyphenols from grape extracts are important aspects of their value as a feed additive in weaned piglets. The aims of this study were to evaluate the presence of polyphenols from grape extracts both in vitro in IPEC cells and in vivo in the duodena and colons of piglets fed a diet containing a 5% solution of grape extracts, and to compare and correlate aspects of their in vitro and in vivo absorption. Total polyphenol content (TPC) and antioxidant status (enzyme activity total antioxidant status [TAS], CAT, SOD, and GPx and lipid peroxidation—TBARS level) were evaluated in the duodena and colons of piglets, which were either given or not given a 5% solution of grape extract. The results of ultraviolet-visible spectroscopy showed that in the cellular and extracellular medium, polyphenols were oxidized (between maximum = 276 nm and maximum = 627 nm) with the formation of o-quinones and dimers. Liquid chromatography–mass spectrometry analysis showed a procyanidine trimer, optionally C2, and a procyanidine dimer as the major polyphenols identified in the grape extract solution. Qualitative evaluation of polyphenols from grape extracts in cells (maximum 287.1 nm) and in the gut (maximum 287.5 nm) as oxidized metabolic products is related to in vitro and in vivo tests. In addition to the presence of polyphenol metabolites, this study shows the presence of unmetabolized procyanidin trimers in the duodenum and tissue of the colon, which is an important point for evaluating the effects of these molecules at the intestinal level. Moreover, an in vivo study showed that a 5% solution of grape extracts in piglets’ diets increased TAS and reduced lipid peroxidation (TBARS) in both the duodenum and the colon, and increased SOD activity in the duodenum and CAT and GPx activity in the large intestine. These parameters are modulated by various polyphenols taken up mainly by procyanidin trimers and catechin on the one hand and metabolites on the other [66]. An overview of studies on the effects of GSSE on the gastrointestinal tract is shown in Table 5.

## 7. Obesity-Related Studies

**Figure 5:**
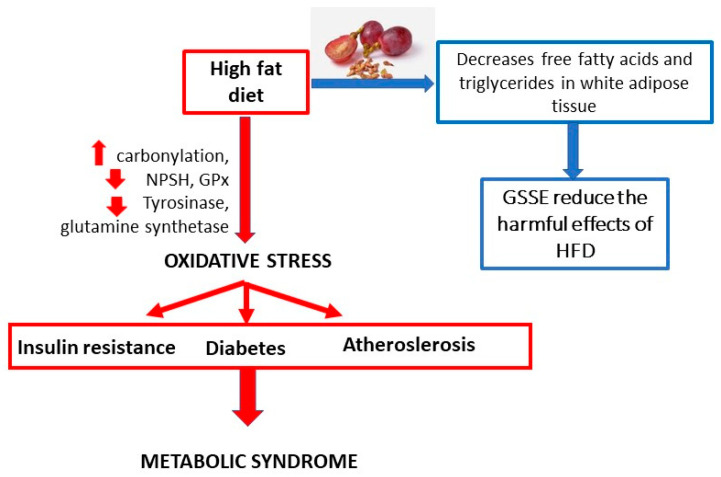
Increased ROS production in accumulated fat contributes to metabolic syndrome.

Obesity, a disease called the 3rd millennium epidemic, is a global problem contributing to morbidity and mortality. The prevalence of adult obesity is 10–25% in most Western European countries and 20–25% in some countries in the Americas. Obesity is a condition in which the natural energy reserve, which is stored in adipose tissue, has risen above normal levels and is harmful to health. This can lead to oxidative stress and chronic inflammation. The affected organs are mainly the heart and liver; however, the brain can also be affected. The effect of a high-fat diet on the values of the biomarkers is shown in Figure 5. GSSE intake helps to decrease free fatty acids and triglycerides in blood and white adipose tissue, and thus reduce the harmful effects of a high-fat diet.

In a study carried out by researchers from the University of Tunisia, attention was focused on the protective effects of grape seed and husk extract on induced oxidative stress in the hearts and livers of *Wistar* rats. High doses of fat were used to induce obesity in rats. Subsequently, individual parameters were monitored in both sexes of rats, namely, increased body weight, increased relative liver weight, and increased relative heart weight. Fat doses caused the accumulation of triglycerides and total cholesterol, especially in the hearts of males and in the livers of both sexes. The conclusion of the study was that treatment with GSE effectively protects the monitored organs disturbed by doses of fat, regardless of gender [67].

Another study [68] analyzed the relationship between oxidative stress and cardiac dysfunction using an experimental model of HFD. The cardioprotective effect of grape seed and husk extract was studied in a rat model. The extract reduced all the harmful effects of HFD. Therefore, it can be stated that GSE has cardioprotective effects and appears to be a safe, natural remedy for obesity [68].

Charradi et al. [69] studied the protective effect of GSSE against oxidative brain damage in obese individuals. They investigated whether sex has an effect on brain damage in obesity, and if so, whether GSSE could have a protective effect. The conclusion of the experiment was that GSSE had the potential to alleviate the detrimental lipotoxic effect of HFD that occurred in the male brain and in the postmenopausal female brain.

In a study carried out by Caimari et al. [70] the beneficial effects of GSE, which was administered in low doses but contained a high proportion of procyanidins, were evaluated. The extract was studied in relation to body weight and fat storage in hamsters. The results showed a significant reduction in body weight gain in hamsters with the addition of GSE to the diet. Reduced weight of all white adipose tissue was noted, and a reduced obesity index was observed. Intake of procyanidin extract reversed the increase in plasma phospholipids induced by a higher-fat diet. Changes have been observed in lipid metabolic pathways. Administration of GSE resulted in lower levels of free fatty acids in plasma and further reduced lipids and accumulation of triglycerides in white adipose tissue. The conclusion of the experiment was that the use of the extract, even at low doses, protects against fat accumulation. Administration of the extract also improved the plasma lipid profile of the experimental animals [70].

Intake of GSSE resulted in the reduction of all the harmful effects caused by the administration of fats. The results indicate the great potential of this extract when used as a safe preparation against lipotoxic agents and as a prevention against fat-induced brain damage [11].

A study conducted by El Ayed [71] investigated the protective effect of GSSE against high fat-induced dishomeostasis in rat lungs. The study investigated the effects of HFD on changes in pulmonary oxidative stress and energy metabolism, and on the presumed protection provided by GSSE. It was characterized by GSSE composition determined by gas chromatography–mass spectrometry. The effect of HFD on the pulmonary oxidation state was also analyzed by assessing the levels of oxidation of lipids, non-protein thiols (NPSH), and superoxide anion. Furthermore, the effects of HFD on creatine kinase (CK), malate dehydrogenase (MDH), and mitochondrial complex IV were evaluated. The results showed that HFD induced pulmonary oxidative stress characterized by increased carbonylation, decreased NPSH, and inhibition of antioxidant activities of enzymes such as glutathione peroxidase. HFD also altered intracellular mediators of the lung, such as superoxide anion, and increased pulmonary xanthine oxidase activity. Furthermore, HFD caused copper and lead depletion from the lungs. HFD also reduced enzyme tyrosinase and reduced glutamine synthetase activity. Moreover, HFD altered pulmonary energy metabolism by increasing CK activity and reducing MDH and mitochondrial complex IV activity. Importantly, all these changes were effectively corrected with GSSE treatment. In conclusion, it can be stated that GSSE has the potential to alleviate the harmful lipotoxic effect of HFD on the lungs.

The aim of the research carried out by Ohiama et al. [72] was to determine whether GSE could protect male *Rattus norvegicus* against obesity caused by high doses of fats. Catechin and epicatechin were analyzed as the main components of the extract. GSE was able to suppress weight gain and adipose tissue weight in a *Rattus norvegicus* model with induced obesity. GSE further improved metabolic abnormalities and affected fatty acid oxidation in the liver. The study concluded that GSE, containing high concentrations of catechin and epicatechin, can improve the health of *Rattus norvegicus* with induced obesity [72].

An interesting study carried out by Charradi et al. [67] showed the great potential of treatment with GSE. The extract effectively protected the monitored organs, which were disrupted by doses of fat regardless of gender. GSE therefore appears to be a cardioprotective and safe remedy for obesity.

The results of a study conducted by Arora et al. [73] showed the effect of GSE on obesity caused by high doses of dietary fat in experimental animals. The study found significant effects on weight loss and food intake in rats given GSE. The results showed that the weights of the liver and adipose tissue were significantly lower in these rats than in those the group of experimental animals that did not receive the extract. Some other parameters, such as total cholesterol, high-density-lipoprotein cholesterol level, and glucose level, were significantly reduced. A histological study found that the lipid contents of the heart and liver were significantly lower in rats fed HFD and GSE than in those fed HFD alone. In conclusion, GSE may be beneficial in suppressing induced obesity [73].

Bashir et al. [74] studied the effect of GSE in combination with cadmium on the fat profiles of experimental rats. The aim was to determine whether GSE can reduce the levels of markers associated with obesity. Table 6 shows the changes in plasma lipid levels in control and experimental animals.

Plasma VLDL is metabolized to cholesteryl ester-enriched intermediate low-density lipoprotein (IDL) and LDL particles via hydrolysis of triglycerides by lipoprotein lipase (LPL) and hepatic lipase (HL). In addition, cholesteryl ester transfer protein (CETP) transfers CE from HDL to VLDL in exchange for triglyceride (TG) to HDL. Therefore, static measurements of cholesterol in the LDL pool (LDL-C) represent the steady state of production of VLDL, its metabolism to LDL, and the receptor-mediated clearance of LDL by the LDL receptor (LDLR) [75].

In a study conducted by Quesada et al. [76] rats were given PACs, which normalized triglycerides and plasma LDL-cholesterol (both parameters increased significantly with HFD) and also led to a tendency to reduce hypercholesterolemia. Gene expression analysis revealed that PACs suppressed both the expression of key hepatic regulators of lipogenesis and the assembly of VLDL, such as SREBP1, MTP, and DGAT2, all of which were overexpressed by HFD. The results of this study are summarized in Table 7.

In Bashir et al.’s [74] study, GSE administration reduced plasma TG levels by 40%, total cholesterol (TC) levels by 13%, and LDL-C levels by 40%. Therefore, GSE is also an effective agent for reducing TG and LDL-C in HFD-associated dyslipidemia.

The aim of the study conducted by Park et al. [77] was to assess the effects of GSE on obesity in C57BL/6J mice. The effects of GSE supplementation on the serum and liver lipid concentrations of this experiment are shown in Table 8. Serum TG was significantly lower in the HFD + GSE group than in the HFD group. Serum TC was significantly higher in the HFD group than in the other groups. The ratio of serum HDL-cholesterol to HDL-cholesterol/TC was significantly higher in the HFD + GSE group than in the other groups. Hepatic TG and TC in the HD + GSE group were significantly lower than in the HFD group.

The conclusion of the study conducted by Park et al. [77] was that GSE has beneficial effects and prevents some of the negative effects of HFD. It has been shown that GSE supplementation reduced weight gain, feed intake, TG, and TC in the serum and liver. The effects of GSE on induced obesity in mice suggest that GSE has potential anti-obesity effects and may alleviate obesity-related symptoms, including hyperlipidemia, cardiovascular disease, and insulin resistance.

The aims of the study carried out by Moreno et al. [78] were to evaluate the effects of GSE on fat-metabolizing enzymes, namely, pancreatic lipase, lipoprotein lipase and hormone-sensitive lipase, and to evaluate its potential use for the treatment of obesity. GSE rich in bioactive phytochemicals showed inhibitory activity on fat-metabolizing enzymes (pancreatic lipase and lipoprotein lipase), suggesting that GSE could be useful as a possible treatment to reduce fat absorption and fat accumulation in adipose tissue.

**Figure 6:**
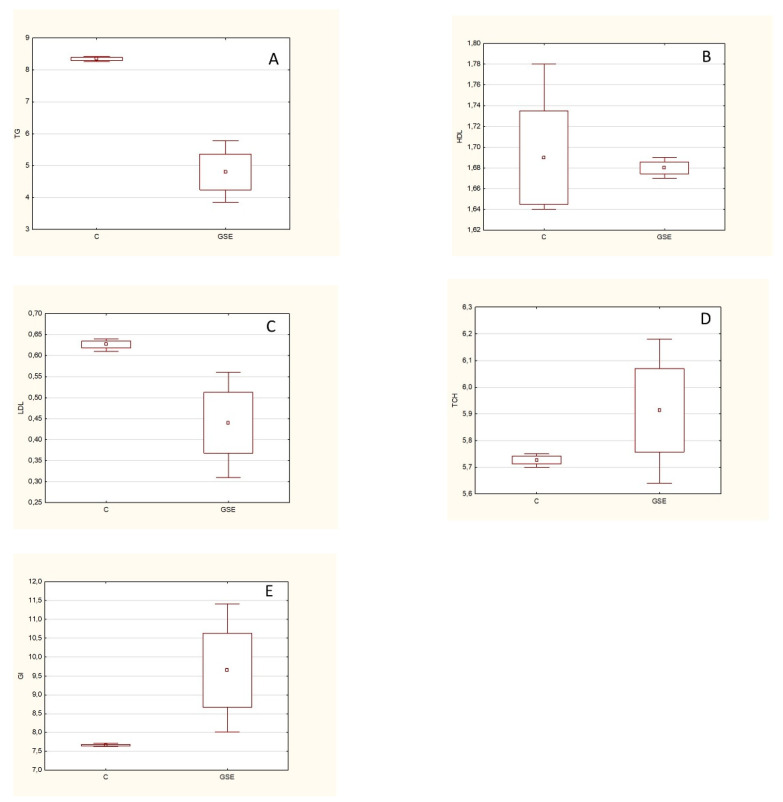
Concentration values: (**A**) TGC, (**B**) HDL, (**C**) LDL, (**D**) TCH, and (**E**) GL. C—control group, GSE—variant with the addition of GSE. The values of biochemical markers are expressed in mmol/L. The results of lipid metabolism showed significant changes in the values of TGC, LDL (*p* < 0.05), and glucose (*p* > 0.05). From these results, it can be stated that the administered extract has a positive effect on these monitored parameters. A: TGC *p <* 0.05; B: HDL *p* > 0.10; C: LDL *p* < 0.05; D: TCH *p* > 0.10; E: GL *p* > 0.05

Zucker rats (fa/fa) were used in a previously unpublished experiment (conducted by the authors of this manuscript). It is a model of spontaneous genetic obesity that exhibits hyperphagia, hypercholesterolemia, hyperinsulinemia, and hyperlipidemia. These animals were given GSE (2 g/day) every day for 6 weeks. Subsequently, the animals were euthanized and markers related to lipid metabolism (triacylglycerides (TGC), HDL, LDL, and total cholesterol (TCH)) were analyzed from the blood plasma, and the glucose content (GL) was determined (Figure 6).

An overview of obesity related studies is shown in Table 9.

## 8. Other Studies

Li et al. [79] investigated the anti-inflammatory effect and the mechanism of action of PACs from grape seeds in mice with induced ear swelling and in mice with carrageenan-induced hind paw oedema. The results showed that 10–40 mg/kg of PACs inhibited carrageenan-induced paw oedema and ear swelling in mice in a dose-dependent manner. Specifically, 10 mg/kg PACs reduced MDA content in inflamed paws; inhibited N-acetyl-beta-D-glucosaminidase (beta-NAG) activity; and reduced nitric oxide content, and radioactive immunoassay contents of IL-1 beta, TNF-alpha, and PGE2 in mice with paw oedema. The inhibitory effect of PACs on all the above indices was more pronounced than the effect of 2 mg/kg dexamethasone. In conclusion, PACs have an anti-inflammatory effect in mice. The mechanisms of the anti-inflammatory effect are relevant for scavenging oxygen free radicals, lipid peroxidation, and inhibiting the production of inflammatory cytokines [79].

Ras et al. [3] studied the effect of GSE on blood pressure in untreated hypertensive patients. Dietary polyphenols, such as grape products, can have beneficial effects on the cardiovascular system, including antihypertensive effects. The study examined the effect of a specific GSE rich in low molecular weight polyphenol compounds on outpatient blood pressure in untreated individuals with pre-hypertension and stage I hypertension. In addition, potential mechanisms that could have an effect on blood pressure and platelet aggregation were investigated. The study was designed as a double-blind, placebo-controlled, randomized, parallel, interventional study involving 70 healthy subjects with systolic blood pressure between 120 and 159 mm Hg. This was followed by an 8-week intervention period, during which subjects consumed capsules containing either 300 mg/day GSE or a placebo (microcrystalline cellulose). Urine and blood samples were taken before and after the period of consummation of capsules, and outpatient blood pressure values were measured. The mean baseline systolic blood pressure was 135.8 (SE 1.3) mm Hg and the mean diastolic blood pressure was 81.5 (SE 0.9) mm Hg. Blood pressure values were slightly affected by GSE polyphenols compared placebo with an effect of −3.0 mm Hg for systolic blood pressure and −1.4 mm Hg for diastolic blood pressure. Vasoactive markers such as endothelin-1, NO metabolites, and asymmetric dimethylarginine; plasma renin activity; and platelet aggregation were not affected by GSE consumption. The results show that consumption of GSE rich in polyphenols does not significantly reduce outpatient blood pressure in untreated individuals with pre-hypertension and stage I hypertension.

Martinez et al. [80] investigated the effect of the addition of GSE on gastrointestinal cleavage in vitro and on the AOC of meat emulsions. GSE was added as an antioxidant to turkey and pork emulsions with mechanically separated meat. AOC and lipid oxidation were measured in controls and emulsions supplemented with 0.5% GSE before and after in vitro gastric digestion. The results showed that gastric digestion increased the AOC of meat 8−11 times. Based on the data obtained, the addition of GSE (0.5%) to meat emulsions is sufficient to prevent lipid oxidation and improve the AOC of the emulsions [80].

Cakir et al. [81] examined grape seeds and their ability to protect the cholestatic liver against ischemia/reperfusion injury. Eighteen *Wistar* rats were divided into three groups. In the control and study groups, cholestasis was ensured by bile duct ligation. Oral administration of 50 mg/kg/day GSE was started 15 days before bile duct ligation and continued until the second operation in the study group. Serum, plasma, and liver samples were taken. Laboratory analysis, tissue glutation, and histopathological examination were performed, and MDA levels and MPO activity were observed. The results showed a significant reduction in hepatic glutathione levels, and significant increases in MDA levels and MPO activity were observed after ischemia/reperfusion injury in cholestatic rats. Serum and plasma levels were also significantly higher in the cholestatic ischemia/reperfusion injury group. Hepatic necrosis and fibrosis were found during histopathological examination. Oral administration of GSE altered all these parameters and histopathological findings, with the exception of serum bilirubin levels. In conclusion, oral treatment with GSE can improve liver function and relieve inflammation and oxidative stress in cholestatic ischemia/reperfusion injury [81].

A study carried out by Enginara [82] investigated the effects of grape extract on lipid peroxidation, its antioxidant activity, and lymphocytes in the blood of rats exposed to X-rays. The study was designed to evaluate supplementary extracts of grapes and vitamin E and their effectiveness in lipid peroxidation. The researcher also evaluated the blood in rats exposed to X-rays. Rats were divided into three groups. The control group (CG) received intraperitoneal (i.p.) physiological serum 1 mL/day (n = 10), the second group received i.p. vitamin E (VG) 50 mg/kg/day (n = 10), and the third group received grape extracts 50 mg /kg/day (n = 10). Four weeks later, rats were dosed with 6 Gy of radiation. Blood samples were taken 24 h after irradiation, and lymphocyte concentrations, MDA, reduced GSH, nitrate, nitrite, reduced ascorbic acid, retinol, beta-carotene, and ceruloplasmin concentrations were analyzed. Concentrations of GSH (*p* < 0.05), retinol (*p* < 0.001), beta-carotene (*p* < 0.05), and ceruloplasmin (*p* < 0.001) were found to be higher in the GSE group than in the control group, while MDA (*p* < 0.001) and nitrite concentrations (*p* < 0.05) in GSE-supplemented rats were lower than in the control group. The results show that GSE was able to increase the antioxidant status and reduce free radical-induced lipid peroxidation in blood samples from rats exposed to X-rays. The antioxidant effect of GSE administered to animals was more greater than that of vitamin E administered before whole body irradiation in rats [82].

Bagchi et al. [83] studied free radicals and PAC extract from grape seeds to determine their importance for human health and disease prevention. Free radicals are involved in more than a hundred disease states in humans, including arthritis, hemorrhagic shock, atherosclerosis, ischemia, Alzheimer’s disease, Parkinson’s disease, gastrointestinal dysfunction, carcinomas, and AIDS. The ability to scavenge free radicals as a function of the concentration or dose of the new IH636 GSPE was assessed in in vitro and in vivo models, and the ability of GSPE to scavenge free radicals was compared with the abilities of with vitamins C and E, and beta-carotene. These experiments showed that GSPE is highly bioavailable and provides significantly greater protection against free radicals, lipid peroxidation, and free radical-induced DNA damage than vitamins C and E and beta-carotene. GSPE has also been shown to be cytotoxic to some human cells. The comparative protective effects of GSPE and vitamins C and E on tobacco-induced oxidative stress and apoptotic cell death in human oral keratinocytes have been investigated. Oxidative tissue damage was determined by lipid peroxidation and DNA fragmentation, while apoptotic cell death was determined by flow cytometry. It was found that GSPE provides significantly better protection than vitamins C and E, both individually and in combination. GSPE has also shown excellent protection against liver and kidney damage by regulating the bcl-X-L gene and possibly reducing oxidative stress. In addition, GSPE has shown excellent protection against ischemic reperfusion injury of the myocardium and myocardial infarction in rats. Furthermore, GSPE application increases the sun protection factor in volunteers. Moreover, GSPE supplementation improves chronic pancreatitis in humans. These results indicate that GSPE provides excellent protection against oxidative stress and free radical-mediated tissue damage [83].

In 2017, Bialek et al. [84] investigated the effects of grape seed and pomegranate oil on fatty acid profiles and cholesterol contents in chickens. The aim of the study was to determine whether supplementing the chicken’s diet with grape seed oil or pomegranate oil would affect the cholesterol content and fatty acid profile of the liver. Ross 308 chickens (*n* = 24) were fed a diet enriched with grape seed oil (group G) or pomegranate oil (group P). The diet of the control group (group C) was based on soybean oil. Modification of chicken nutrition with grape seed oil and pomegranate oil affected the fatty acid profile in the liver. The presence of punic acid (cis-9, trans-11, cis-13 C18: 3) was detected. Furthermore, a significant amount of folic acid (cis-9, trans-11 C18: 2), the main isomer of conjugated linoleic acids, was found. Its natural sources in the diet are ruminant meat and milk; however, the addition of pomegranate oil and grape oil to chicken feed caused a significant increase in the fatty acids in the liver. This proves that punic acid is efficiently converted to rhomic acid. Grape seeds and pomegranate seeds, therefore, seem to be interesting feed supplements.

A study conducted in 2018 [85] investigated the effects of supplementing the diet with grape seeds on performance, carcass traits, plasma biochemistry, antioxidant status, and ileal microflora in broilers. Experimental diets included a diet without additives (control group) and three types of diet with the addition of grape seeds (10, 20, and 40 g/kg diet). The experiment lasted for 42 days. The addition of 20 g/kg to the basal diet increased final body weight and weight gain, improved the feed conversion ratio, and did not affect the feed intake. The addition of 20 g of grape seeds significantly (*p* < 0.05) increased the percentage of carcass yield. However, the addition of 40 g significantly reduced the percentage of abdominal fat in broilers. The physical properties of the meat and the chemical composition were not significantly affected by the modifications. Plasma protein, albumin, globulin, AST, and ALT concentrations were not significant in the grape seed groups compared with the control group. Grape seed broilers had lower levels of glucose, total lipids, triglycerides, and cholesterol compared with control broilers (*p* < 0.05). The addition of 40 g significantly (*p* < 0.05) increased the activities of reduced GSH, catalase, superoxide dismutase, and glutathione peroxidase. The ileal pH was not significantly affected. Broilers fed a diet supplemented with grape seeds had lower amounts of ileal *Streptococcus spp*. and populations of *Escherichia* coli, but higher amounts of *Lactobacillus spp.* (*p* < 0.05). No adverse effects on the health of broilers due to the use of grape seeds were identified. Therefore, they could be recommended as an addition to the broiler diet to improve performance, reduce blood lipids, increase AOC, and reduce harmful bacteria in the ileum [85].

## 9. Conclusions

Results of studies on the effects of GSSE confirm that a high dose of GSSE is a safe and effective antioxidant that can be further tested in clinical experiments for long-term maintenance of renal function. In diabetes mellitus, serious secondary complications are nephropathy, neuropathy, and retinopathy. Results of the studies also showed that oral feeding of grape seeds significantly alleviated renal dysfunction and pathological changes in diabetic mice.

The effects of PACs on the development of diabetic nephropathy in E-deficient mice were studied, and it was shown that in a group of mice for which PACs were added to the diet, high blood cholesterol levels were significantly reduced. Furthermore, renal function was restored, and this led to a reduction in albuminuria compared with the untreated group.

The anti-inflammatory, antiapoptotic, and pro-proliferative effects of *Vitis vinifera* L. seed extract on the livers of rats with induced type 2 diabetes mellitus were also confirmed. It has been found that the consumption of grapevine seeds improves the overall condition of the liver in rats with this disease. Oxidative stress and associated hyperlipidemia and hyperglycemia play important roles in the development of diabetic nephropathy.

Among other things, extracts of plant origin (including GSE) have inhibitory effects on alpha-amylase and alpha-glucosidase activity. These are important enzymes for the digestion of starch in humans.

In rabbits with high cholesterol, the effect of PAC-rich GSE, which alleviates the development of aortic atherosclerosis, was investigated. There were reductions in LDL-cholesterol and malondialdehyde when rabbits were fed with PAC-rich extract.

Moreover, grape seed procyanidins protect endothelial cells from peroxynitrite damage and improve endothelial relaxation in the human artery, providing new evidence for cardioprotection.

Regarding the use of GSSP extracts for the treatment of cancer, cytotoxicity and apoptotic cell death induced by extracts from the skins and seeds of *Vitis vinifera* L. on A431 skin cancer cells were investigated. Phytochemicals from *Vitis vinifera L*. can selectively target cancer cells and serve as potential antitumor agents providing better efficacy in killing cancer cells.

Furthermore, the chemopreventive potential of blue grape marc powder against colorectal cancer in HT-29 cells was investigated. The study evaluated the antiproliferative and antigenotoxic effects of grape marc powder against cancer of the prostate, colon, liver, and tongue.

Most of the protective biological effects of resveratrol are associated with its antioxidant, anti-inflammatory, and antiapoptotic properties and other indirect pathways. The continuing public interest and the growing availability of resveratrol on the market require an overview of available in vitro and in vivo experiments. Although resveratrol has shown potential for the treatment of stroke in laboratory animals and in in vitro studies in human cells, there is still a need for research in preclinical conditions. This review summarizes the findings on the neuroprotective potential of resveratrol in stroke with a focus on in vitro and in vivo experimental models and some proposed mechanisms of action. Dementia is a risk factor for obesity since fat can adversely affect the brain. GSE and peel can provide protection against high lipid dietary steatosis and increased oxidative stress. GSSE could therefore be used as a safe preparation against lipotoxic agents and as a preventative against brain damage caused by higher doses of lipids.

The gastrointestinal tract plays a central role in the absorption, distribution, metabolism, and excretion of flavonoids, which ultimately defines the health effects of these bioactive substances. These aspects are modulated by the interaction of flavonoids with other components of the diet, environmental factors, and hosts and microorganisms of the gastrointestinal tract. Flavonoids can protect the intestinal epithelium against pharmacological and food toxins, modulate the activity of enzymes involved in the absorption of lipids and carbohydrates, maintain the integrity of the intestinal barrier, modulate intestinal hormone secretion, modulate the immune system of the gastrointestinal tract, and develop potential anticarcinogenic activity in the intestine. PACs with a degree of polymerization greater than three remain unabsorbed in the gastrointestinal tract and accumulate in the intestinal lumen. Accordingly, the gastrointestinal tract can be considered a key organ for health. Moreover, PACs have antimicrobial effects that disrupt cariogenic or ulcerogenic pathogens in the mouth and stomach. An overview of these studies is shown in Table 10.

## Figures and Tables

**Table 1:** Results of studies focused on the effects of grape seed and skin extract (GSSE) on diabetes mellitus.

Monitored Extract/Compound	Effect on Tissue/Organism	Health Effect	Model of Study
GSE/flavanols [25]	effect on renal endothelial function	improve the condition of patients with microalbuminuria	human
GSE/polyphenols [33]	effects on insulin	influencing neuroregulatory factors, nerve signalling pathways	human
GSE/catechin 3-gallates [34]	inhibitory effects on alpha-amylase and alpha-glucosidase activity	effect on enzymes for starch digestion in humans	human
GSE/PAC [27]	anti-inflammation	reduce periodontal inflammation and alveolar bone loss	rats
GSSE [28]	oxidative stress/renal dysfunction	effectively protect against all the harmful effects of diabetes, such as renal dysfunction	rats
GSE [30]	anti-inflammatory/antiapoptotic	improve the overall liver condition	rats
GSE/polyphenols [35]	Improve the lipid profile	alleviate dyslipidemia and ocular complications, reduce risk of atherogenicity	rats
GSE [36]	the effects on serum amylase levels/antiapoptopic effect	positive effect on the pancreas	rats
GSE [21]	antioxidant properties	prevented the progression of diabetic nephropathy	rats
GSSE [37]	reduce oxidative stress	alleviating urethral dysfunction	rats
GSE [21,22]	Antioxidant activity	alleviate albuminuria and renal sclerosis in diabetic nephropathy	rats
GSE [23]	alleviate oxidative stress/ inhibiting lipid peroxidation/restoring endothelial function	reducing the risk of vascular disease in patients with diabetes mellitus	rats
GSE/PAC [8]	reduce oxidative stress	reduce blood cholesterol levels, restoring renal function, reduction in albuminuria	mice
GSE [26]	neuroprotective effect	treat diabetic peripheral neuropathy	mice
GSE [29]	anti-diabetic nephrophaty	alleviated renal dysfunction and pathological changes	mice
Grape seed oil [24]	antiapoptotic activity	reduce the apoptosis of pancreatic beta cells	in vitro

Note: GSE—grape seed extract, GSSE—grape seed and skin extract, PAC—proanthocyanidine.

**Table 2:** Results of studies focused on the effects of GSSE on cardiovascular diseases.

Monitored Extract/Compound	Effect on Tissue (Organism)	Health Effect	Model of Study
GSE/flavonoids [42]	antioxidant/anti-inflammatory effects	cardioprotective effects	human
GSE/PAC [44]	protect endothelial cells from peroxynitrite damage/improve endothelial relaxation in the human artery	protective effect due to their specific binding to the outer surface of endothelial cells	human
GSE/PAC [40]	reduce the development of aortic atherosclerosis	reduction in low density lipoprotein cholesterol and MDA, significantly reduce atherosclerosis	rabbits
GSE [41]	reduce lipoprotein peroxidation	against atherosclerosis, prevention of cardiovascular disease.	in vitro

Note: GSE—grape seed extract, PAC—proanthocyanidine.

**Table 3:** Results of studies focused on the effects of GSSE on oncological diseases.

Monitored Extract/Compound	Effect on Tissue (Organism)	Health Effect	Model of Study
GSE [46]	antioxidant activity	reduce the negative impact of chemotherapy	human
GSE/PAC [56]	antiproliferative effects	inhibit the proliferation, migration and invasion of lung cancer	human
GSE [54]	decreased oxidative stress/decreased regulation of histone deacetylase activity/ antiinflammation	anticarcinogenic properties on liver cancer	rats
GSE/polyphenols [47]	antitumor activity/reduces lipid peeroxidation	not effective on Breast cancer, reduces lipid peroxidation induced by doxorubicin treatment.	mice
GSE [51]	induce apoptosis	against colorectal cancer	mice
GSE/PACs [52]	antineoplastic mechanisms	against lung cancer	mice
GSE [45]	cytotoxicity and apoptosis	potential antitumor agents, better efficacy in killing cancer cells	in vitro
GSE/blue grape marc powder (seedless variant, seeds alone) [48]	chemopreventive potential/antiproliferative/antigenotoxic effects	chemopreventive potential in colorectal cancer	in vitro
GSE/resveratrol [49]	antioxidant activity	against human prostate cancer cells	in vitro
GSE/PAC [50]	antiproliferative effects	against oral cancer cells	in vitro
GSE,/PAC [53]	chemopreventive/antiproliferative	against colon cancer	in vitro
GJE/phenolic compounds [55]	chemopreventive/ antitumor	against colon cancer cells and their potential to metastasise	in vitro
GSE rich in chitosan-microencapsulated extracts [57]	chemopreventive/antitumor	antitumor effect due to increased cell interaction.	in vitro

Note: GSE—grape seed extract, PAC—proanthocyanidine, GJE—grape juice extracts.

**Table 4:** Results of studies focused on the effects of GSSE on the nervous system.

Monitored Extract/Compound	Effect on Tissue (Organism)	Health Effect	Model of Study
GSSE [11]	reduce oxidative stress	against dementia as a risk factor accompanying obesity	rats
GSE [59]	Induce quantitative changes in specific proteins	neuroprotective effect	rats
GSSE [60]	modulation of inflammation/neurotransmitters/reduce oxidative stress	neuroprotective and nephroprotective effect	*in vitro*

Note: GSE—grape seed extract, GSSE—grape seed and skin extract.

**Table 5:** Results of studies focused on the effects of GSSE on the gastrointestinal tract.

Monitored Extract/Compound	Effect on Tissue (Organism)	Health Effect	Model of Study
GSE/grapefruit/PAC [62]	antioxidant effects	protect the gastrointestinal tract by acting on absorption cells and enterohormone-secreting cells	rats
GSE [64]	reduces oxidative stress and fibrosis	against oxidative liver damage and fibrosis caused by biliary obstruction	rats
polyphenols from grape marc [66]	antioxidant activity/reduce lipid peroxidation	protect the gastrointestinal tract	in vitro, piglets
Grape and cranberry extracts [63]	effect of microbial tissue on polyphenols	pronounced antimicrobial effect, especially against *Bacteroides*, *Prevotella* and *Blautia coccoides-Eubacterium rectale*.	*in vitro*

Note: GSE—grape seed extract, PAC—proanthocyanidine.

**Table 6:** Effects of GSE on cadmium-induced changes in the plasma lipid profiles of control and experimental rats (Bashir et al. [74]).

Biomarkers	Control (mg/100 mL)	GSE (dose 100 mg/kg bw) (mg/100 mL)	Cadmium (Cd) (dose 5 mg/kg bw) (mg/100 mL)	Cd+GSE (dose 5 mg/kg bw + 100 mg/kg bw) (mg/100 mL)
**Total cholesterol**	88.18 ± 1.39 ^a^	86.10 ± 2.16 ^b^	132.24 ± 6.27 ^d^	100.34 ± 4.49 ^c^
**Triglycerides (TG)**	76.28 ± 1.17 ^a^	74.09 ± 1.08 ^b^	80.60 ± 5.49 ^d^	63.05 ± 4.18 ^c^
**Phospholipids (PL)**	110.14 ± 4.32 ^a^	107.30 ± 0.01 ^b^	154.92 ± 8.54 ^d^	130.91 ± 7.12 ^c^
**Free fatty acids (FFA)**	98.19 ± 6.27 ^a^	94.13 ± 0.27 ^b^	145.29 ± 8.78 ^d^	97.79 ± 6.83 ^c^
**LDL-C**	20.14 ± 1.34 ^a^	18.09 ± 2.01 ^b^	71.82 ± 4.32 ^d^	45.72 ± 7.50 ^c^
**HDL-C**	43.76 ± 2.16 ^a^	46.51 ± 2.4 ^b^	24.45 ± 4.45 ^d^	35.33 ± 3.21 ^c^
**VLDL-C**	7.18 ± 0.32 ^a^	5.89 ± 1.01 ^b^	15.24 ± 0.07 ^d^	8.89 ± 0.02 ^c^

Note: Compared with control rats, significantly (*p* < 0.05) increased levels of plasma cholesterol, TG, FFA, and PL were observed in Cd-treated rats. In rats treated with GSE and Cd, plasma cholesterol, TG, FFA, and PL levels were significantly (*p* < 0.05) reduced compared with rats treated with Cd alone. Significant (*p* < 0.05) increases in LDL-C and VLDL-C and a significant (*p* < 0.05) decrease in plasma HDL-C levels in Cd-treated rats were observed compared with control rats. In rats treated with high-dosage GSE, plasma LDL-C levels and VLDL-C were significantly (*p* < 0.05) reduced, and HDL-C levels were significantly (*p* < 0.05) increased compared with rats supplemented with Cd. LDL—low density lipoprotein, VLDL—very low density lipoprotein, HDL—high density lipoprotein.

**Table 7:** Plasma lipid levels in rats fed a standard diet, a high-fat diet, and a high-fat diet supplemented with proanthocyanidin extract (Quesada et al. [76]).

Biomarkers	Control (mg/100 mL)	HFD Group (mg/100 mL)	HFD + GSE Group (mg/100 mL)
**Triglycerides**	107.3 ± 10.6	204.0 ± 2.3	129.4 ± 12.3
**Total cholesterol**	57.9 ± 2.8	95.9 ± 5.7	83.5 ± 4.5
**HDL cholesterol**	35.6 ± 7.9	60.6 ± 4.1	51.0 ± 4.9
**LDL cholesterol**	3.5 ± 0.1	15.2 ± 2.0	6.6 ± 1.0
**HDL–C/LDL–C ratio**	8.7 ± 2.2	4.0 ± 0.6	7.0 ± 0.11
**Total C/HDL–C ratio**	1.4 ± 0.03	1.6 ± 0.15	1.7 ± 0.11
**Free fatty acids**	20.5 ± 2.1	22.9 ± 2.0	14.3 ± 1.1

Note: GSE—proanthocyanidin extracts from grape seeds; HFD—high-fat diet.

**Table 8:** Serum and liver lipid concentrations (Park et al. [77]).

Biomarkers	Normal Diet (ND) (mg/100 mL)	High Fat Diet (HFD) (mg/100 mL)	HFD + GSE (mg/100 mL)
**Serum**			
**Triglycerides**	54.39 ± 4.02 ^c^	94.07 ± 7.36 ^a^	79.17 ± 3.91 ^b^
**Total cholesterol**	139.28 ± 5.70 ^c^	176.97 ± 7.88 ^a^	158.75 ± 17.09 ^b^
**HDL-cholesterol**	74.48 ± 3.7 ^b^	58.88 ± 6.33 ^b^	85.19 ± 5.44 ^a^
**Liver**			
**Triglycerides**	28.15 ± 4.27 ^b^	37.04 ± 5.07 ^a^	32.37 ± 3.64 ^b^
**Total cholesterol**	6.73 ± 0.40 ^b^	8.98 ± 0.73 ^a^	5.37 ± 0.58 ^c^

**Table 9:** Results of obesity related studies.

Monitored Extract/Compound	Effect on Tissue (Organism)	Health Effect	Model of Study
GSSE [69]	against oxidative brain damage	alleviate the detrimental lipotoxic effect of HFD that occurred in the male brain and in the postmenopausal female brain	human
GSE [70]	alleviate the detrimental lipotoxic effect of HFD	reduce free fatty acids in plasma and reduce lipids and accumulation of triglycerides in white adipose tissue	hamsters
GSE [77]	reduce serum and liver lipid concentrations	alleviate obesity-related symptoms, including hyperlipidemia, cardiovascular disease, and insulin resistance	mice
GSSE [67]	reduce oxidative stress in the heart and liver	protects the monitored organs disturbed by doses of fat	rats
GSSE [68]	reduce oxidative stress/cardiac dysfunction	reduced all the harmful effects of HFD	rats
GSSE [71]	reduce oxidative stress/energy metabolism	alleviate the harmful lipotoxic effect of HFD on the lungs	rats
GSE/catechin and epicatechin [72]	improve metabolic abnormalities/affected fatty acid oxidation in the liver	improve the health with induced obesity	rats
GSE [73]	reduce total cholesterol, HDL cholesterol and glucose levels	beneficial in suppressing induced obesity	rats
GSE/PAC [74,76]	normalised triglycerides and plasma LDL-cholesterol	reduce hypercholesterolemia	rats
GSE [78]	effect on fat-metabolising enzymes,	treatment to reduce fat absorption and fat accumulation in adipose tissue.	in vitro

Note: GSE—grape seed extract, GSSE—grape seed and skin extract, PAC—proanthocyanidine.

**Table 10:** Results of other GSSE studies.

Monitored Extract/Compound	Effect on Tissue (Organism)	Health Effect	Model of Study
GSE/polyphenols [3]	effect on blood pressure	GSE rich in polyphenols does not significantly reduce outpatient blood pressure in untreated individuals with pre-hypertension and stage I hypertension	human
GSE/PAC [79]	anti-inflammatory effect/ reduce lipid peroxidation	inhibited carrageenan-induced paw oedema and ear swelling in mice	mice
GSE [81]	antioxidant activity/anti-inflammatory	improve liver function and relieve inflammation and oxidative stress in cholestatic ischemia/reperfusion injury	rats
GSE [82]	antioxidant activity/reduce lipid peroxidation	alleviate negative effect of X-rays	rats
GSE/PAC [83]	antioxidant activity	protection against liver and kidney damage by regulating, against ischemic reperfusion injury of the myocardium and myocardial infarction	rats, in vitro
GSE and pomegranate oil [84]	effect on fatty acid profile	affected the fatty acid profile in the liver	chickens
GSE [85]	antioxidant activity/effect on ileal microflora	No effect on health, could be recommended as an addition to the broiler diet	broilers
GSE [80]	antioxidant activity/reduce lipid oxidation	prevent lipid oxidation and improve antioxidant activity	in vitro

Note: GSE—grape seed extract, PAC—proanthocyanidine.

## Abbreviations

ALT	alanine aminotransferase-tissue damage biomarker
AOC	antioxidant capacity
AST	Aspartate aminotransferase-tissue damage biomarker
CAT	catalase-biomarker for oxidative stress
CaCo2 cells	colon cancer cells
CRC	colorectal cancer cells
GPx	glucose peroxidase
GSE	grape seed extract
GSH glutathione	redox biomarker
GSPE	grape seed proanthocyanidin extract
GSSE	grape seed and skin extract
GJEs	grape juice extracts
HFD	high-fat diet
HDL	high density lipoprotein
IC50	inhibitory concentration
LDH	lactate dehydrogenase-tissue damage biomarker
LDL	low density lipoprotein
LP	leucoselect phytosome
MDA malondialdehyde	lipid peroxidation marker
MPO	myeloperoxidase-biomarker for cardiovascular disease risk
NO-cGMP	nitric oxide-cyclic guanosine monophosphate
NPSH	non-protein sulfhydryl
PAC	proanthocyanidine
PCR	polymerase chain reaction
ROS	reactive oxygen species
SOD	*superoxide dismutase*-biomarker for oxidative stress
*TBARS*	thiobarbituric acid reactive substances-*biomarker* of oxidative stress
TSCC	tissue cell carcinoma
